# Diamond Nanoparticles Suppress Migration of T98G Glioblastoma Cells by Targeting ECM–Integrin Interactions and Intracellular Signaling, Leading to Extensive Proteome Alterations

**DOI:** 10.2147/NSA.S540050

**Published:** 2025-11-04

**Authors:** Katarzyna Zawadzka, Barbara Wójcik, Malwina Sosnowska-Ławnicka, Marta Kutwin, Sławomir Jaworski, Agnieszka Ostrowska, Michał Pruchniewski, Mateusz Wierzbicki

**Affiliations:** 1 Department of Nanobiotechnology, Institute of Biology, Warsaw University of Life Sciences, Warsaw, 02-786, Poland

**Keywords:** glioblastoma, diamond nanoparticles, proteomics, migration, integrins, T98G

## Abstract

**Introduction:**

Glioblastoma (GBM) is a highly heterogeneous and aggressive tumor characterized by rapid growth and therapy resistance. The dynamic interactions of tumor cells with the extracellular matrix (ECM) contribute to treatment inefficacy. While diamond nanoparticles (NDs) are emerging as potential antitumor agents, their mechanisms remain incompletely understood. In this study, we investigated spherical NDs with distinct surface compositions and hydrocolloidal stability and their role in regulating crucial cellular processes in T98G glioblastoma cells.

**Methods:**

Two types of detonation diamond nanoparticles (NDs) were characterized using TEM imaging and hydrocolloidal stability assessment in various diluents. Their effects on T98G glioblastoma cells were examined through SEM imaging, cytotoxicity assays, monitoring of spontaneous and collective migration, and early adhesion examination combined with an extensive integrin-blocking panel. Furthermore, characterization via mass spectrometry provided deeper insight into how physicochemical differences between the two NDs types modulate glioblastoma microenvironment and cell responses.

**Results:**

NDs were observed to be both intensively internalized by cells and bound to cell membrane, influencing cellular interactions with the extracellular environment. NDs significantly reduced T98G glioblastoma cell migration within 48 hours and impaired early adhesion by effectively blocking α/β integrins. Modified NDs (NDM) demonstrated enhanced hydrocolloidal stability and stronger integrin blocking efficiency. Proteomic analysis revealed that NDs downregulated proteins involved in RNA processing, splicing, and translation while upregulating ECM-related proteins, which profile changed depending on the NDs type.

**Conclusion:**

These findings suggest that NDs with distinct surface properties may interact with multiple surface receptors, independently modulate intracellular signaling pathways, and remodel the tumor microenvironment by altering ECM protein composition, positioning them as versatile, multi-targeting agents with antitumor potential.

## Introduction

Glioblastoma (GBM) is one of the most lethal and treatment-resistant tumors, accounting for nearly half of all central nervous system malignancies.^1^ The median overall survival of patients diagnosed with glioblastoma and under treatment is approximately 12 months.^2^ Surgical tumor removal is not feasible in every case. Following surgery, adjuvant therapy with radiation and temozolomide can improve patient outcomes and quality of life in the initial months, but it only marginally increases long-term survival rates.^3^

Despite two decades as the standard treatment, temozolomide with radiation remains the primary therapy. Newer approaches, including antiangiogenic agents, TTFields, kinase inhibitors, and personalized immunotherapies (checkpoint inhibitors, CAR-T cells), show promise but have yet to significantly improve GBM outcomes.^4–6^ GBMs are highly heterogenous tumors, which presents a major challenge for achieving long-term therapeutic success. The specific dynamics and remodeling of the tumor extracellular matrix (ECM) contribute to the limited effectiveness of current therapies. Increased ECM stiffness and higher levels of proteins such as thrombospondins, fibronectin, hyaluronan, tenascins, chemokines, and Ca²⁺ ionic imbalance, along with glioma-specific integrin expression – mainly αv and β1 subunits – often impair drug penetration and promote pro-oncogenic pathways.^7–9^

In aggressive and heterogeneous tumors, both collective and single-cell migration, which contribute to metastasis and tumor expansion, are complex yet crucial to investigate.^10^ Another critical membrane-associated process, adhesion to ECM components, plays a fundamental role in tumor mass formation and therapy responsiveness.^11^,^12^ Given the ECM’s role in regulating these processes, targeting its components has gained significant attention as a therapeutic strategy.^13^ Nevertheless, modulating endocytosis-mediated signaling or directly blocking specific cell-surface receptors may also have distinct consequences on tumor progression as the composition of the surfaceome varies between cells.^14^,^15^

T98G, studied in this work, is a well-known glioblastoma grade IV cell line widely used in drug screening.^16^ The common use of this cell line in research is due to great resemblance of the cells phenotype, glycolysis metabolism and invasive characteristics in both 2D and 3D models to primary glioblastoma cells isolated directly from patients and suggested as a reference cell line.^17^,^18^ Notably, T98G cells are resistant to both temozolomide, the standard chemotherapeutic agent for glioblastoma, and to radiation therapy.^19–21^ Given that therapy resistance remains one of the major challenges in glioblastoma management, employing cells with these characteristics provides a relevant model for exploring novel therapeutic strategies.^22^

Diamond nanoparticles (NDs), known for their high systemic biocompatibility, have demonstrated potential as antitumor agents with tumor mass reduction observed in glioblastoma xenograft models.^23–25^ Detonation NDs have also been shown to successfully cross the blood–brain barrier in mice. They exhibit anti-angiogenic activity, showing the strongest effect among carbon allotropes such as graphene, graphite, multiwalled nanotubes, and fullerenes.^26^ NDs have further been shown to interfere with pro-oncogenic signaling pathways.^27^,^28^ The smaller size of detonation NDs compared with NDs synthetized by high-pressure-high-temperature (HPHT) method, along with their complex surface, rich in functional groups, that can increase the affinity for certain extracellular proteins, such as growth factors, positions detonation NDs as valuable agents in nanomedicine-related research.^29^ Their ease of functionalization facilitates the formation of nanoparticle-drug complexes, which exhibit high targeting efficacy, prolonged action, and reduces side effects, compared with drugs administrated alone.^30^,^31^ The surface composition of NDs and the proportion of surface carbon with sp^2^ hybridization have been shown to influence cellular interaction, biocompatibility and inflammatory response in rats.^32^ Nevertheless, the precise mechanisms of ND action remain unclear as their effects appear to vary depending on tumor type, cell line, and the physicochemical characteristics of the nanoparticles used.^33^

In this study, we hypothesized that NDs can reduce glioblastoma invasiveness by modulating cell migration and adhesion through interactions with both the cell surface and ECM. Given the impact of nanoparticle surface properties on cellular responses and ECM interactions, we employed two types of detonation-derived NDs in these experiments. Specifically, we investigated whether NDs influence ECM protein binding and integrin-mediated signaling, thereby affecting tumor cell motility. Integrin profiling was conducted to analyze receptor availability for pro-oncogenic signaling. Eventually, a comprehensive proteomic analysis of T98G cells was performed to examine the downstream effects of integrin blockade and potential intracellular mechanisms following NDs treatment.^34^

## Methods

### Physicochemical Stability of Nanoparticles Hydrocolloids

Diamond nanoparticle powders were obtained commercially from US Research Nanomaterials (Houston, USA) as pure ND and diamond nanoparticles functionalized with -COOH groups (NDM – nanodiamond modified). Suspensions of 1000 mg/L in ultrapure water were sonicated for 5 minutes with 20 Hz amplitude and 30-second breaks after every minute of sonication. Nanoparticles were diluted to 50 mg/L in ultrapure water, cell culture medium EMEM (ATCC; Manassas, VA, USA), and cell culture medium EMEM (ATCC) supplemented with 10% fetal bovine serum (FBS, Gibco, Thermo Fisher Scientific, Waltham, MA, USA). The samples were analyzed immediately after diluting the nanoparticles stock and after 24 hours at 37°C. Before the measurements of Zeta Potential with Smoluchowski approximation in the Nano-ZS90 Zetasizer (Malvern Instruments, Malvern, UK) samples were stabilized at room temperature for 2 minutes. Each group was measured in triplicate with 4 measurements per replicate. For hydrodynamic diameter (Z-Ave) and polydispersity index (PDI), nanoparticles were diluted to 10 mg/L, centrifuged at 2000 RPM for 2 minutes, and measured in the same time points as above. Each triplicate of measurements counted 3 readings. Results are presented as mean values with a standard deviation.

### TEM

ND and NDM hydrocolloids of 20 mg/L were air-dried on copper grids and visualized using JEM-1220 transmission electron microscope (JEOL, Tokyo, Japan) at 80 KeV with a Morada 11-megapixel camera (Olympus Soft Imaging Solutions, Münster, Germany).

### Cytotoxicity

T98G cells were purchased from American Type Culture Collection (ATCC). The culture was maintained in EMEM (ATCC) supplemented with 10% FBS (Gibco, Thermo Fisher Scientific) and 1% penicillin/streptomycin mix (1000 µg/mL) as recommended by the manufacturer. For cytotoxicity tests, cells were seeded at a concentration of 1×10^4^ cells per well of 96-well plate 24 hours before the experiment. Nanoparticles were prepared in EMEM without FBS supplementation in a final concentration of 50 mg/L. ND and NDM at 500 mg/L were mixed with cell culture medium at a ratio of 1:10. In the control group, medium was diluted with ultrapure, sterile water. Right before the treatment, the cells were washed with PBS to remove the remains of FBS. Cells were incubated with nanoparticles for 4 hours and 24 hours. After the incubation, the LDH leakage was measured using Pierce CyQuant LDH Cytotoxicity Assay in accordance to the manufacturer protocol. Results are presented as a percentage of cytotoxicity and calculated by dividing the difference between the test sample and spontaneous LDH leakage control (negative control) by the difference between maximum LDH leakage and spontaneous negative control. For the Presto Blue assay (PB, Thermo Fisher), cells were prepared in the same manner. At the end point of each incubation variant, 11 µL PB was added to each well, and after 2 hours of incubation, fluorescence intensity was measured using an excitation wavelength of λ = 560 nm and an emission wavelength of λ = 590 nm. Data are presented as a percentage relative to the control group after blank signal subtraction. Morphology images were taken after 24-hour incubation with nanoparticles in FBS-free medium using an inverted microscope (Olympus Soft Imaging Solutions, Münster, Germany).

### SEM

T98G cells were cultured on round, pre-treated cover glasses in a 24-well plate. Cover glasses were soaked in 1M HCl for 24 hours then rinsed at least 3 times in ultrapure water and autoclaved. T98G cells were seeded at 8×10^4^ per well in 1 mL of FBS-supplemented EMEM. After 24 hours, the cells were washed with PBS and treated with nanoparticles prepared by diluting the 10-times concentrated (500 mg/L) suspension in cell culture medium by adding 10% of the total volume (1 mL). The control medium contained sterile water as 10% of the total volume. Cells were incubated under standard conditions. Samples were fixed after 4 hours and 24 hours of incubation with nanoparticles. Cells were thoroughly washed and fixed with 2.5% glutaraldehyde at 4°C overnight. After rinsing from glutaraldehyde residues, the samples were contrasted for 45 minutes with 1% osmium tetroxide. Subsequently, the cells were washed 3 times with PBS and dehydrated in an ethyl alcohol gradient. Eventually, cover glasses were coated with a gold layer and imagined on the Quanta 200 microscope (FEI, Hillsboro, OR, USA).

### Cell Adhesion

To evaluate the influence of diamond nanoparticles on the adhesion capacity of T98G glioma cells, 96-well tissue culture plates were partially coated with collagen type I from rat tail (Gibco, Thermo Fisher Scientific) and BSA solution, which served as a negative control. BSA was inactivated at 80°C for 15 minutes and sterile-filtered. Collagen solution was prepared from a 3 mg/mL stock solution in 0.2 mM sterile-filtered acetic acid. All steps were conducted on ice. Plates were incubated overnight at 4°C covered from light then washed 3 times with DPBS. A day before the experiment, the T98G cells were seeded on cell culture flasks in a fully supplemented EMEM medium. On the day of the experiment, the cell culture medium was discarded, cells were thoroughly washed with PBS, and treated with diamond nanoparticles (ND and NDM) prepared as above in FBS-free EMEM. Incubation was conducted for 4 hours. Due to the potential impairment of integrin function by trypsin detachment, cells were first incubated with 4 mM EDTA in HBSS for 15 minutes then detached by scraping. Collected cells were centrifuged on 200 × g for 6 minutes, washed, and centrifuged again. Single-cell suspensions were calculated, and after adjusting the concentration, the cells were seeded at 1.5×10^4^ per well in 100 µL of cell culture medium on pure plastic, BSA-coated surface and collagen I-coated surface. Measurements were performed at 3 time points: 15 minutes, 30 minutes, and 60 minutes since seeding. Cells were seeded on different plates, each for a particular time point. At each time point, the culture medium was discarded, wells were gently washed with PBS to remove unattached cells, and plates were frozen dry. Positive controls remained in medium throughout the experiment. After the final time point, all plates were processed together. Green fluorescent stain CyQuant GR (Thermo Fisher Scientific) diluted 1:400 in PBS was added as 50 µL per well and incubated for 10 minutes in the dark. Subsequently, the remaining stain solution was discarded, and the cells were gently washed and lysed with lysis buffer. After an additional 10-minute incubation, fluorescence intensity was measured with excitation at λ = 480 and emission at λ = 520 nm. The assay was conducted twice with at least five replicates per group.

### Live Cell Migration

T98G cells were seeded on the 8-well chamber slide (Ibidi, Germany) at 4.5×10^4^ cells per well in 300 µL cell culture medium. Three hours before the analysis, the cells were stained with Hoechst 33342 (Invitrogen Thermo Fisher Scientific) for 10 minutes then washed carefully and left to regeneration. ND and NDM at 50 mg/L were prepared in cell culture medium EMEM supplemented with 10% FBS and 25 mM HEPES and were introduced to the cells 1 hour before the beginning of imaging. Time-lapse imaging was conducted using an FV1000 confocal microscope with a motorized stage (Olympus Corporation, Japan) at 10× magnification. Slides were incubated in a temperature-controlled compartment at 37°C (Solent Scientific, UK) equipped with a CO_2_ chamber (Pecon, Germany) to maintain optimal physiological conditions. Images were captured at 5-minute intervals for 24 hours and analyzed using the TrackMate plugin in ImageJ.^35^ For each sample, a minimum of four image stacks were captured. The mean median velocity, mean total distance traveled, and total distance traveled per number of tracks were analyzed for tracks with a duration time exceeding 23 hours.

### Migration Assay

T98G cells were seeded on a 12-well plate (Thermo Scientific) inside Ibidi silicon inserts (Ibidi) at 2×10^4^ cells per chamber in 70 µL of complete EMEM. After 24 hours, inserts were removed, and cells were washed twice with PBS. Cells were then treated with ND and NDM (50 mg/L) in FBS-free EMEM, prepared by diluting 10% of the total volume with nanoparticle suspension. In control groups, culture medium was diluted in the same manner using ultrapure, sterile water. Images of migration were captured at three locations per well during the first hour of the experiment using an inverted light microscope (Leica TL-LED, Wetzlar, Germany) with a Leica MC190 HD digital camera and LAS V4.10 software (Leica). After 24 hours, the medium was replaced with standard EMEM supplemented with 10% FBS. The final images were taken after 48 hours. Images were analyzed manually using ImageJ software.^36^ Results are presented as the percentage of the remaining cell-free area at the end of the experiment.

### Integrins Panel

To identify particular integrins presented on a T98G cell’s surface, the α/β Integrin-mediated Cell Adhesion Array Combo Kit colorimetric (ECM532, CHEMICON, Sigma Aldrich) was used. The kit contains two 96-well plates that enabled performing the analysis of cells treated with ND and NDM at 50 mg/L in at least three replicates per group. The assay was conducted according to the attached handbook. The kit for paneling integrins expression contained two plates coated with antibodies against commonly detected integrins α/β: α1, α2, α3, α4, α5, αV, αVβ3, and a negative control on a plate α and β1, β2, β3, β4, β6, αVβ5, α5β1, with a negative control on a plate β.

Plates were rehydrated shortly before use. Cells were detached from the flasks by non-enzymatic agents – 4 mM EDTA in HBSS for 20 minutes. Due to strong adhesion, cells were scraped, centrifuged for 3 minutes at 200 × g, washed twice with 2 mM EDTA in DPBS, and centrifuged after each wash. Cells were counted and seeded at 3×10^4^ per well. Plates were incubated for 2 hours in a cell culture incubator. After incubation, unattached cells were gently washed out with PBS. Plates were washed three times, and staining solution was added for 5 minutes. Wells were thoroughly rinsed, and extraction buffer was added to release the stain. After 5 minutes, OD was measured at λ = 560 nm.

### Proteomics Analysis

Proteomic analysis was conducted on T98G glioblastoma cells treated with 50 mg/L of ND and NDM in FBS-free EMEM for 24 hours. One day before the experiment, cells were seeded in 75 cm² tissue culture flasks at 6×10^5^ cells per flask, reaching ~70% confluency at the experiment’s start. Each experimental group included three replicates. After incubation, cells were carefully washed with PBS and collected by scraping. Cell pellets were centrifuged three times (200 × g, 6 minutes), each time resuspended in fresh PBS. After the final wash, samples were snap-frozen in liquid nitrogen and stored at −80°C until further processing. Samples for mass spectrometry (MS) analysis were prepared using Mini MS Sample Prep Kit (cat. A40006; ThermoFisher Scientific) following the manufacturer’s instructions. Total protein concentration in cell lysates was obtained with Pierce 660nm Protein Assay Kit (cat. 22662; ThermoFisher Scientific). The volumes of samples containing 100 µg of total protein were designated for further processing, which included reduction of disulfide bridges, alkylation of free sulfhydryl groups, and enzymatic hydrolysis with a mixture of enzymes, trypsin and Lys-C. The resulting peptides were purified using C18 solid-phase extraction cartridges then dried in a vacuum concentrator and resuspended in LC-MS grade water containing 0.1% (v/v) formic acid. Peptide concentration in every sample, after measurements with Pierce Quantitative Fluorometric Peptide Assay (cat. 232900, ThermoFisher Scientific), was adjusted to 0.8 µg/µL. Proteomic analysis was performed using an Orbitrap Exploris 480 mass spectrometer coupled to a Neo Vanquish liquid chromatograph. Quality control (QC) samples were prepared by mixing 5 µL from each tested sample with the control sample. The list of detected and defined proteins including the changes in the expression levels was composed via Proteome Discoverer 3.0 based on the SwissProt Homo Sapiens database using the following filters: abundance ratio variabilities [%]: has any value in every ratio; found in Samples: has any value in at least 3 samples; protein FDR (false discovery rate) confidence: has at least level high in protein FDR confidence combined. Proteins with an expression fold change (FC) ≥ 2 at *p-value* <0.05 compared to the untreated control group were selected for further investigation.

### Functional Annotation and Pathways Analysis

The functional annotation clustering was performed with the Database for Annotation, Visualization, and Integrated Discovery (DAVID) online software.^37^ To reduce annotation redundancy, the functional clustering tool identified the most enriched proteins within biological processes (BP), cellular component (CC), and molecular function (MF) categories of gene ontology (GO). The algorithm is based on identifying the groups of similar annotations according to Kappa statistics. Clusters were generated based on Modified Fisher’s Exact p-value (EASE Score <0.01) with classification stringency parameters set as follows: similarity term overlap = 4; similarity threshold = 0.75; multiple linkage threshold = 0.75. FDR cutoff was marked on the value = 0.05. For downregulated proteins in the ND group, CC and MF enrichment analysis was performed without clustering, using the same EASE Score and FDR cutoffs. For pathways analysis, the EASE score threshold was relaxed to <0.05. Protein network interactions were visualized using STRING (Search Tool for the Retrieval of Interacting Genes/Proteins) database with a FULL network at a high confidence score (≥0.7).^38^ Networks from STRING were imported into Cytoscape 3.10.3 and labelled with log2FC (fold change) values.^39^ For pathway enrichment analysis, KEGG (Kyoto Encyclopedia of Genes and Genomes), REACTOME, and WIKI pathways databases were searched; EASE score 0.05 was implicated and FDR cutoff value stayed at 0.05. Additional data visualization was performed using GraphPad Prism and SRplot.^40^ The complete list of proteins used for GO analysis and lists of proteins contributed to every demonstrated GO category are attached in the supplementary data (see Figures S1–S3 and Table S1).

### Statistical Analysis

Statistical analysis for migration assays was conducted using one-way ANOVA with Duncan’s post hoc test. For other analyses, two-way ANOVA or mixed-effects models were applied (for adhesion assays). Statistically significant differences are marked with asterisks, symbols, or compact letters above bars, depending on the analysis method used. All images were analyzed using ImageJ software. Statistical parameters for proteomics analysis are described in the Proteomics Analysis section.

## Results

### FBS in Cell Culture Medium Prevents Excessive Aggregation of Diamond Nanoparticles

**Figure 1:**
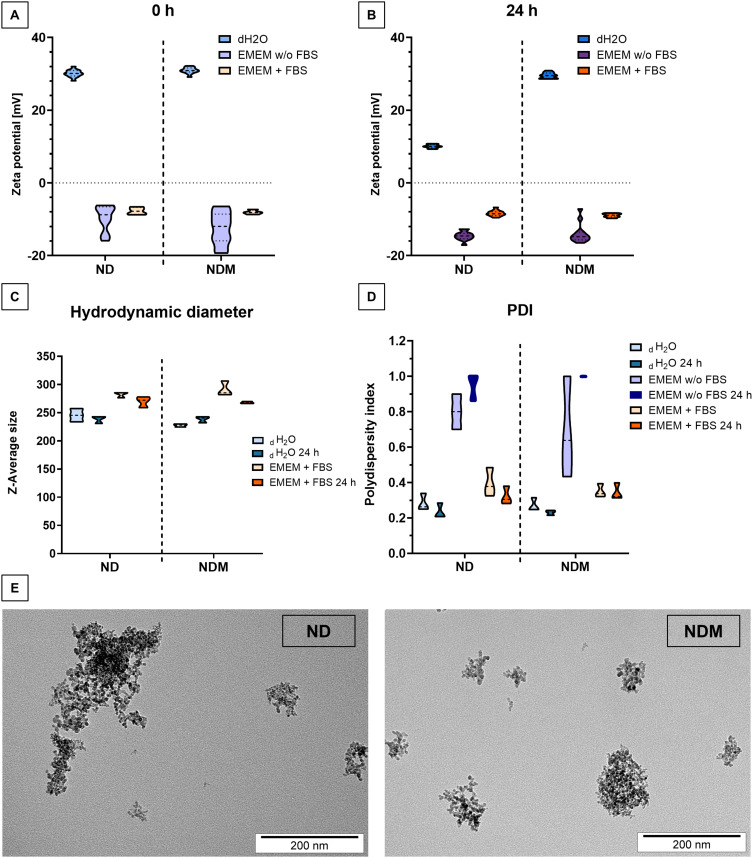
FBS in cell culture medium prevents excessive aggregation of diamond nanoparticles. Physicochemical characterization of diamond nanoparticles in different dispersants: (**A**) Zeta potential measured immediately after the preparation (n=4); (**B**) Zeta potential measured after 24 hours incubation (n=4); (**C**) Hydrodynamic diameter for diamond nanoparticles dispersed in ultrapure H_2_O and FBS-supplemented EMEM measured at the preparation time and after the 24 hours incubation (n=3); (**D**) PDI for diamond nanoparticles dispersed in ultrapure water, FBS-free EMEM, and FBS-supplemented EMEM at the preparation time and after the 24 hours incubation with data presented as medians with quartiles; (**E**) TEM images of ND and NDM.

**Abbreviations**: ND, diamond nanoparticles; NDM, modified diamond nanoparticles; EMEM w/o FBS, serum-free EMEM; EMEM + FBS, FBS-supplemented EMEM; PDI, polydispersity index.

Zeta potential (ZP) and hydrodynamic diameter were measured to assess the stability of diamond nanoparticles in different diluents for cell culture. Diamond nanoparticles suspended in ultrapure water, regardless of type, exhibited ZP values close to +30 mV (Figure 1A). After 24 hours, only NDM maintained its ZP while ND values dropped to +10 mV (Figure 1B). In both culture media, the ZP of nanoparticles became negative. In FBS-supplemented EMEM, the ZP was −7.3 mV for ND and −8.1 mV for NDM, which remained stable after 24 hours of incubation. A similar trend was observed for NDM in FBS-free EMEM with ZP values of −12.5 mV immediately after preparation and −13.9 mV after 24 hours. Only ND in FBS-free EMEM showed a decrease in ZP, changing from −9.7 mV to −14.6 mV after 24 hours.

Hydrodynamic diameter measurements revealed aggregates averaging 245 nm for ND and 227 nm for NDM in water suspensions (Figure 1C). These results, along with PDI values, remained stable over 24 hours of incubation (Figure 1D). In FBS-supplemented EMEM, aggregate sizes were on average 73 nm larger than in water suspension. ND was more polydispersed with a PDI of 0.39 compared to 0.29 in water-dispersed ND. A similar trend was observed for NDM with an average hydrodynamic diameter of 314.9 nm in FBS-supplemented EMEM, 78 nm larger than in water suspension, and PDI values of 0.35 for cell medium and 0.28 for hydrocolloid. Both parameters decreased over time for both types of diamond nanoparticles in FBS-supplemented medium, although PDI remained above 0.3. However, nanoparticles in FBS-free EMEM were highly polydispersed (PDI >0.8), making cumulative analysis impossible.

TEM visualization of nanoparticles revealed the presence of individual particles demonstrating the tendency to form aggregates (Figure 1E).

The nanoparticles were also characterized using Raman spectroscopy. The results, presented in our previous study,^33^ identified ND as ND-nf and NDM as ND-COOH. Raman spectra measurements showed a diamond peak at 1320–1321 cm^−^¹ for both nanodiamonds. Additionally, ND demonstrated an intense G-band at 1572 cm^−^¹ compared to NDM G-band at 1562 cm^−^¹.

### Diamond Nanoparticles are Efficiently Internalized by the T98G Glioblastoma Cell Line without Causing Significant Toxicity

**Figure 2:**
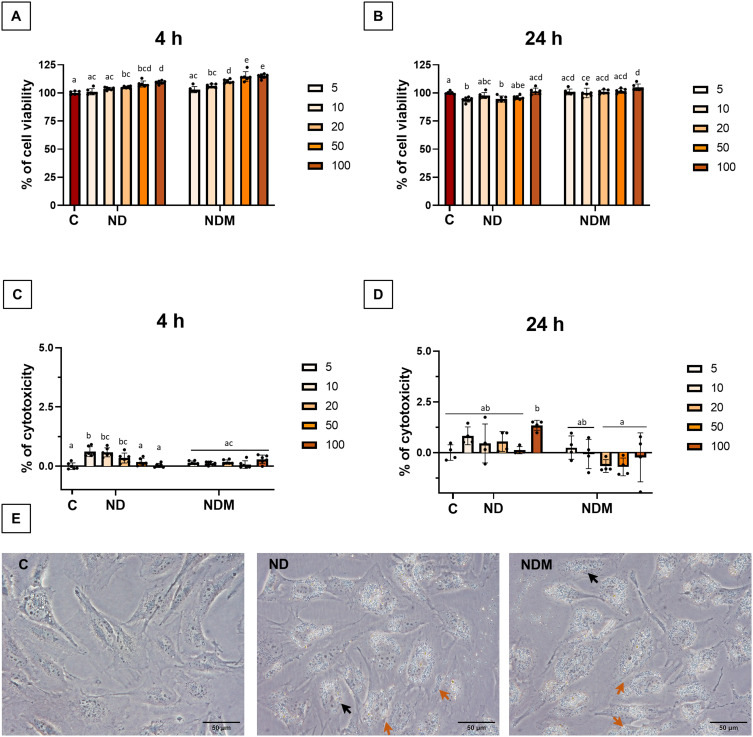
Diamond nanoparticles cause no direct toxic effect to T98G. Toxicity of ND and NDM at 5, 10, 20, 50, and 100 mg/L towards T98G glioblastoma cells assessed by: (**A**) Cell viability evaluation with Presto Blue after 4 hours of incubation with nanoparticles (n=6); (**B**) Cell viability evaluation with Presto Blue after 24 hours of incubation with nanoparticles (n=6); (**C**) Membrane integrity analyzed by LDH Pierce Assay after 4 hours incubation with nanoparticles (n=4); (**D**) Membrane integrity analyzed by LDH Pierce Assay after 24 hours incubation with nanoparticles (n=4); results presented in (**A**–**D**) were analyzed by standard two-way ANOVA with multiple pairwise comparison; statistical differences between groups are indicated by the superscript letters a-e above the bars; *p* ≤ 0.05; (**E**) Morphology of T98G after 24 hours with nanoparticles at 50 mg/L taken unDer Standard inverted microscope with 40× magnification; Black arrow indicates nanoparticle aggregates in the perinuclear area, while the Orange arrow indicates aggregates at the cell–cell contact area; scale bar = 50 µm.

**Abbreviations**: C, control; ND, diamond nanoparticles; NDM, modified diamond nanoparticles.

Cell morphology, metabolic activity, and membrane integrity were evaluated at two time points: 4 hours and 24 hours post-exposure to nanoparticles. Metabolic activity of T98G cells was slightly enhanced during the first 4 hours of incubation in a concentration-dependent manner (Figure 2A). A statistically significant increase was observed for ND at concentrations ≥20 mg/L and NDM at concentrations ≥10 mg/L, although the differences did not exceed 15%. After 24 hours, the highest metabolic activity increase was 5% above control levels for 100 mg/L NDM (Figure 2B). LDH release measurements did not indicate any membrane disruption regardless of nanoparticle type or concentration (Figure 2C and D).

**Figure 3:**
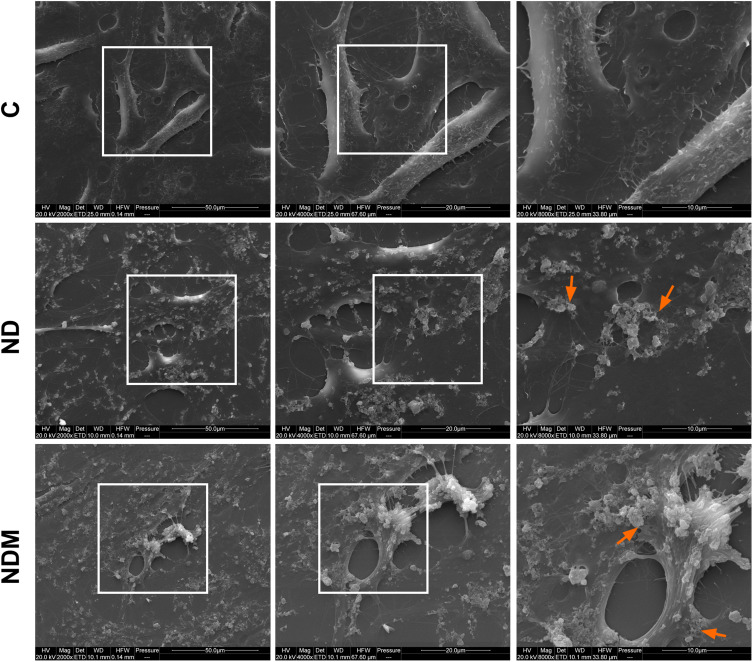
SEM images of T98G cells after 4 hours treatment with diamond nanoparticles. Images taken under the three magnifications; white squares indicate the image fragment that is magnified in the next image. Arrows indicate accumulated NDs.

**Abbreviations**: C, control; ND, diamond nanoparticles; NDM, modified diamond nanoparticles.

**Figure 4:**
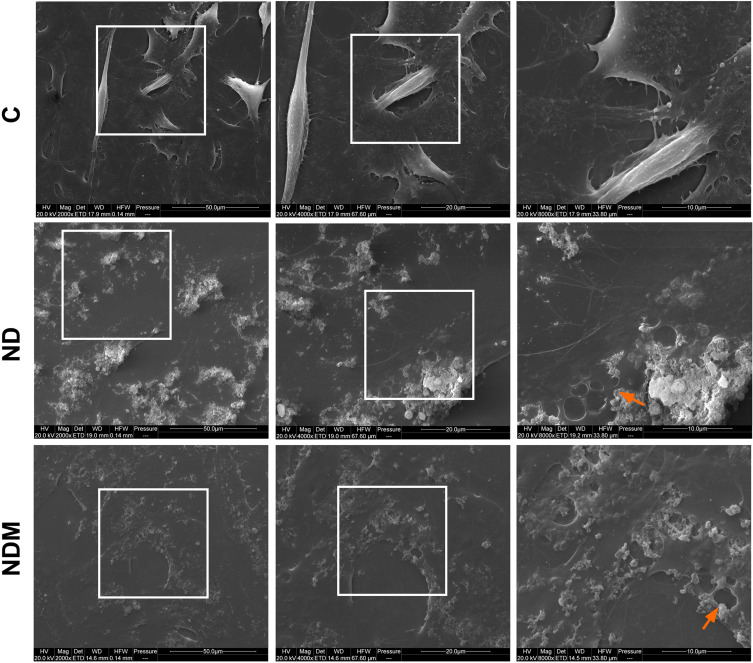
SEM images of T98G cells after 24 hours treatment with diamond nanoparticles. Images taken under the three magnifications; white squares indicate the image fragment that is magnified in the next image. Arrows indicate accumulated NDs.

**Abbreviations**: C, control; ND, diamond nanoparticles; NDM, modified diamond nanoparticles.

Optical microscopy confirmed no morphological changes in treated cells compared to control, as well as nanoparticle accumulation in perinuclear and cell–cell contact areas (Figure 2E, arrows). Electron microscopy visualized nanoparticle aggregates interacting with cells. Intercellular contact areas showed dense nanoparticle accumulation (Figure 3, arrows). Despite high aggregation, including some aggregates >1 µm, internalized nanoparticles were observed within 4 hours, with some bound to cell membranes. Cells developed elongated protrusions, and retraction fibers were visible following the trailing cell edge (Figure 4). Additionally, numerous membrane invaginations and fully formed endocytic vesicles were observed surrounding aggregates (Figure 4, arrows). Cell morphology remained unchanged across treatment groups.

### NDs Impair Migration of T98G Cells During Prolonged Exposure

**Figure 5:**
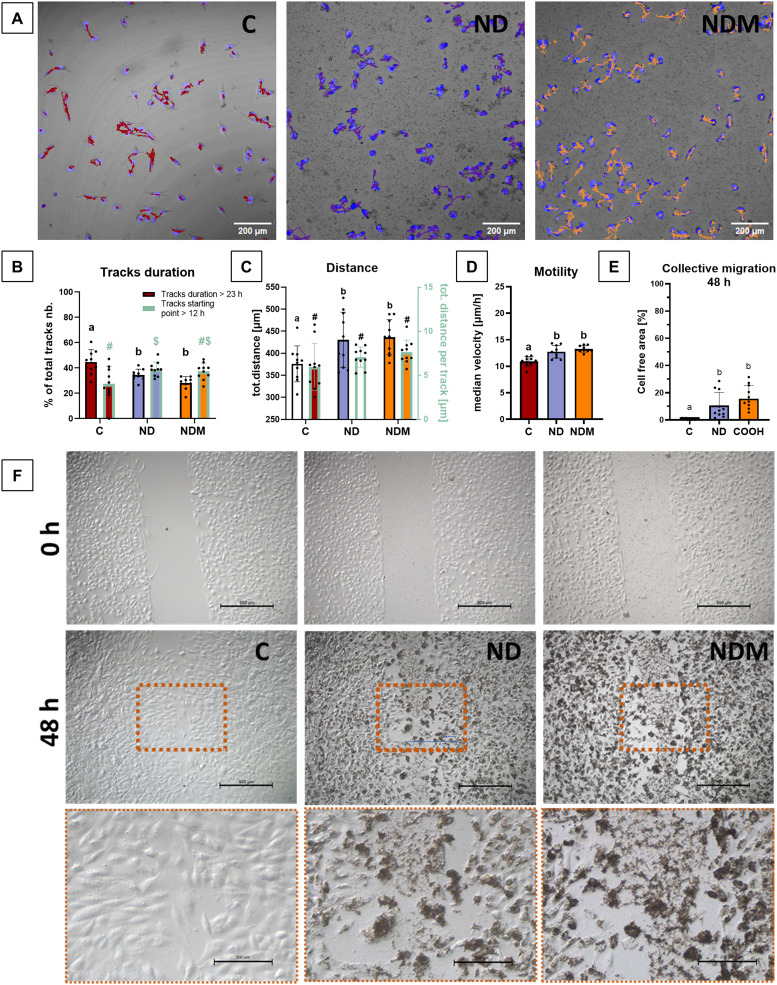
Diamond nanoparticles decreased the collective cell migration. (**A**) T98G time-lapse images after singular tracks analysis in TrackMate in ImageJ; whole tracks recorded in 24 hours are labelled with coloured lines; scale 200 µm; (**B**) tracks with duration above 23 hours and tracks with starting point after 12th hour of incubation divided per total number of tracks in the field of view; for each parameter standard one-way ANOVA was performed; statistical significance is indicated by compact letters above black-bordered bars (duration time) and different symbols above green-bordered bars (tracks starting point), respectively; n=10; (**C**) Total distance travelled measured for tracks with duration time above 23 hours (left y-axis) and divided per number of tracks above 23 hours (right y-axis); for each parameter standard one-way ANOVA was performed; statistical significance is indicated by compact letters above black-bordered bars (tot. distance) and different symbols above green-bordered bars (tot. distance per track), respectively; n=10; (**D**) Median velocity calculated in “TrackMate” in ImageJ; results are presented as mean values with a standard deviation; statistical significance is indicated by compact letters above bars (one-way ANOVA); n=9; (**E**) Percentage of cell-free area after 48 hours incubation in migration assay; calculated in ImageJ; one-way ANOVA; statistical differences between groups are indicated by the superscript letters a, b above the bars; *p* ≤ 0.05; (**F**) Images from under the inverted microscope at the beginning of the migration assay and after 48 hours; scale bar indicates 500 µm; zoomed areas exposing overloaded cells in the migrating front are marked with dotted line.

**Abbreviations**: C, control; ND, diamond nanoparticles; NDM, modified diamond nanoparticles; tot. distance, total distance; total tracks nb., total tracks number.

Time-lapse imaging let us observe the unrestricted movement of T98G from one hour after introducing the nanoparticles to 24 hours of incubation. All cells demonstrated short paths, quick and chaotic movements, and not well-established moving patterns and taxis (Figure 5A). Analysis of long-duration tracks (>23 hours) indicated a decrease in track number following treatment (Figure 5B) with a higher proportion of shorter migration paths emerging in the second half of incubation. The total distance travelled remained comparable across groups (Figure 5C), however the median velocity increased from 10.91 µm/h to 12.72 µm/h and 13.25 µm/h after ND and NDM treatment (Figure 5D). The migration assay, based on maintaining a cell-free gap within the monolayer, was conducted over 48 hours to assess cell movement in the presence of nanoparticles. Diamond nanoparticles decreased the T98G migration rate. After 48 hours, the cell-free area was 10% larger in ND-treated cells and 15% larger in NDM-treated cells compared to controls (Figure 5E). Cells at the leading migration front were swollen and overloaded with nanoparticles (Figure 5F).

### NDs Alter Cell Adhesion via Multi-Integrin Blockage

**Figure 6:**
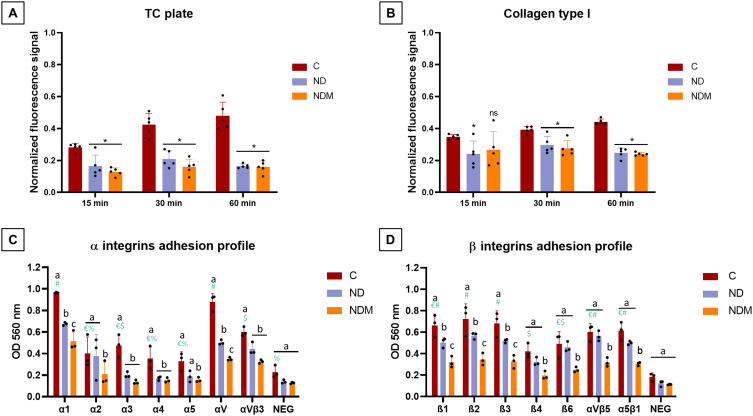
Diamond nanoparticles inhibit α and β integrin-mediated adhesion based on their surface chemistry. (**A**) Fluorescence measured after washing the wells and staining the cells with green GR stain (Thermo Scientific) on the TC plate; (**B**) Fluorescence measured after washing the wells and staining the cells on the collagen I coated plate; (**A** and **B**) Standard two-way ANOVA, n=5, as there were no significant differences between treatment variants [*] indicate statistically significant differences in comparison to control group based on Tukey’s multiple comparisons test (p<0.05); ns – not significant; (**C**) Diamond nanoparticles influence on the distribution of α integrins on the T98G surface; (**D**) Diamond nanoparticles influence on the distribution of β integrins on the T98G surface; average OD measured according to the manufacturer; standard two-way ANOVA; statistical significance between treatment variants effects within each integrin group based on Tukey’s post hoc is designated by different letters above bars (p<0.05); significant differences between integrins level in the control samples based on Tukey’s post hoc are designated by different symbols above bars (p<0.05); n=3.

**Abbreviations**: C, control; ND, diamond nanoparticles; NDM, modified diamond nanoparticles; TC plate, tissue culture plate; NEG, negative control; OD, optical density.

After evaluation of the diamond nanoparticles affinity to cellular membranes, the next step was to investigate the effect of this dynamic interaction on membrane-dependent processes, such as adhesion to different surfaces. BSA coating was used as a negative control. Cell adhesion followed a linear time-dependent pattern in controls, more pronounced on TC plastic than on collagen-coated surfaces. However, nanoparticle-treated groups exhibited significantly reduced adhesion: 33% lower adhesion on TC plastic (Figure 6A) and 19.5% lower adhesion on collagen-coated plates (Figure 6B). This anti-adhesive effect intensified over time with no significant difference between ND and NDM.

To further investigate ND-mediated adhesion impairment, an α/β integrin profile was analyzed. Highly abundant integrins in T98G cells (OD >0.6) included integrins α1, αV, and αVβ3 (Figure 6C). Both NDs demonstrated the ability to block the integrin-mediated adhesion. NDs significantly reduced α1, α3, α4, αV, and αVβ3 activity. Additionally, NDM significantly restricted adhesion via α2 and α5 subunits and had a more prominent effect on α1 and αV presence. Among β-integrins, prevalent proteins included β1, β2, β3, αVβ5, and αVβ1 (Figure 6D). NDM effectively blocked β integrins, halving the number of adhered cells across all tested types, whereas ND specifically inhibited adhesion via β1, β2, and β3 integrins.

### NDs Affect the RNA Binding Protein Expression While Augmenting the ECM-Related Proteins

**Figure 7:**
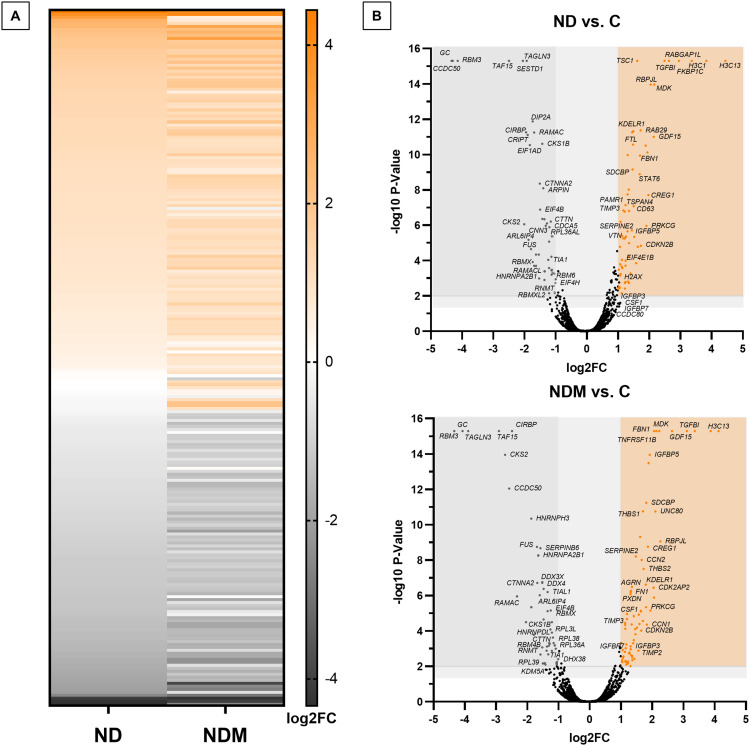
Proteomics analysis performed in DAVID. Graphs created in GraphPad Prism and SRplot. (**A**) Heat map of complete proteomics analysis comparing log2FC of protein abundance referred to untreated control sample between two NDs; (**B**) volcano plots of significantly changed proteins level after ND and NDM treatment; p≤0.05 cutoff is marked with light gray space; gray colored dots represent downregulated protein and Orange dots represent upregulated proteins meeting log2FC>1 and *p*≤0.01 cutoffs; brown-colored dots indicate proteins, which log2FC was equal 1.

Since the overall cellular response and cytotoxicity of ND treatment did not significantly differ between the two types of diamond nanoparticles (ND and NDM), a proteomic analysis was performed to investigate possible molecular alterations. Mass spectrometry (MS) analysis detected 6085 proteins in the tested samples (Figure 7A). This dataset was used for gene ontology (GO) cluster enrichment analysis. The analyzed gene lists included 54 downregulated and 75 upregulated proteins following NDM treatment and 54 downregulated and 68 upregulated proteins following ND treatment. The abundance levels of identified proteins were significantly different from the control with a p-value <0.01 and an FC threshold ≥ 2 (log2FC = 1). These protein levels met both cutoff thresholds. Considering all proteins, which log2FC of abundance level were equal or above |1| there were 71 upregulated and 57 downregulated proteins in ND treated group and 92 highly abundant and 76 downregulated in NDM (Figure 7B and Table S1).

**Figure 8:**
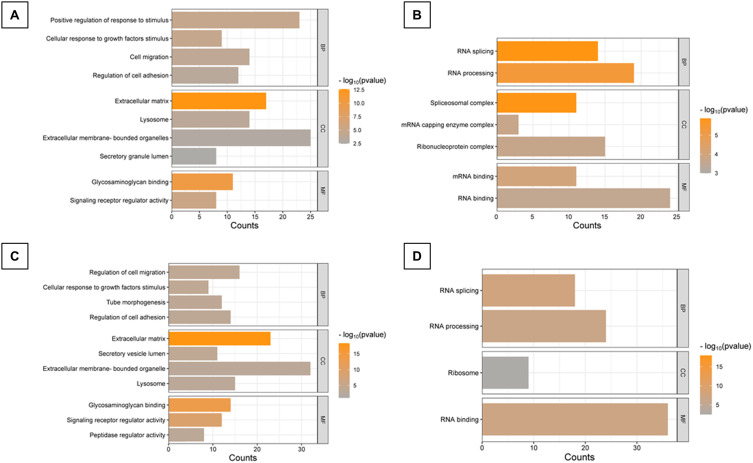
GO analysis of T98G proteins after ND and NDM treatment. (**A**) GO categories for upregulated proteins in T98G cells after ND treatment; (**B**) GO categories for downregulated proteins in T98G cells after ND treatment; (**C**) GO categories for upregulated proteins in T98G cells after NDM treatment; (**D**) GO categories for downregulated proteins in T98G cells after NDM treatment; Set parameters: Similarity Term Overlap = 4; Similarity Threshold = 0.75; Multiple Linkage Threshold = 0.75; EASE score = 0.01; FDR cutoff = 0.05.

**Abbreviations**: ND, diamond nanoparticles; NDM, modified diamond nanoparticles; log2FC, log2 fold change; BB, biological process; CC, cellular component; MF, molecular function; Count, genes on the list.

In both ND- and NDM-treated groups, proteins with a log2FC ≥ |1| were highly enriched in biological processes (BP) GO terms related to cellular responses to stimuli, cell migration, regulation of cell adhesion, and response to growth factor stimuli (Figure 8A, C and Table S2). The cellular component (CC) category was nearly identical in both groups with proteins enriched in lysosomes, secretory granule lumens, extracellular membrane-bounded organelles, and the extracellular matrix, which was the most enriched GO term. The molecular function (MF) of detected proteins corresponded to previously mentioned BP with the most enriched GO terms being glycosaminoglycan binding and signaling receptor regulator activity, both of which were significantly enriched in both treatment variants. Particular proteins detected and assigned to GO categories were listed in the Supplementary Data (Figures S1 and S2).

Among downregulated proteins, the majority were involved in RNA processing with 19 proteins contributing to this category in the ND-treated group (Figure 8B) and 24 in the NDM-treated group (Figure 8D). Considering additional enriched GO terms in the NDM group ribosomal proteins were the only cellular components involved, whereas in the ND group, altered proteins were associated with the spliceosomal complex, mRNA capping enzyme complex, and ribonucleoprotein complex. The highest enrichment score ES=4.78, described as (-log10(p-value)), was linked to the spliceosomal complex, indicating significant alterations in RNA processing pathways. In both groups, the most enriched molecular function was RNA binding, with more than 20 proteins categorized under this function. Specifically, in ND-treated cells, mRNA binding was particularly enriched.

**Figure 9:**
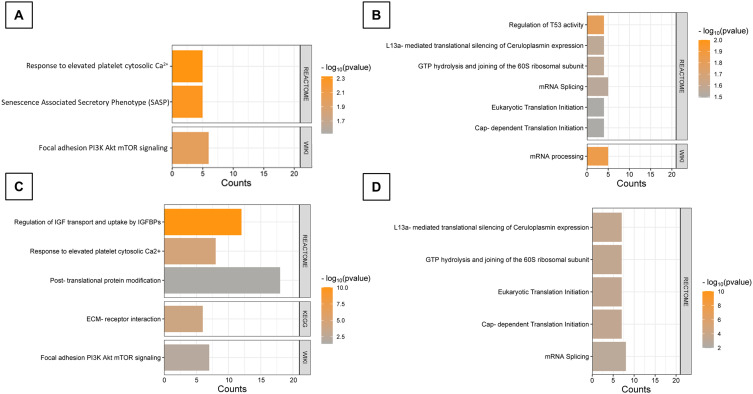
Signaling pathways contributing to altered proteins. Comparison of the results from three main pathways data bases: WIKIPATHWAYS, REACTOME_PATHWAYS, KEGG_PATHWAY. (**A**) Results for upregulated proteins in ND treated group; (**B**) Results for downregulated proteins in ND treated group; (**C**) Results for upregulated proteins in NDM treated group; (**D**) Results for downregulated proteins in NDM-treated group.

**Abbreviations**: ND, diamond nanoparticles; NDM, modified diamond nanoparticles.

**Figure 10:**
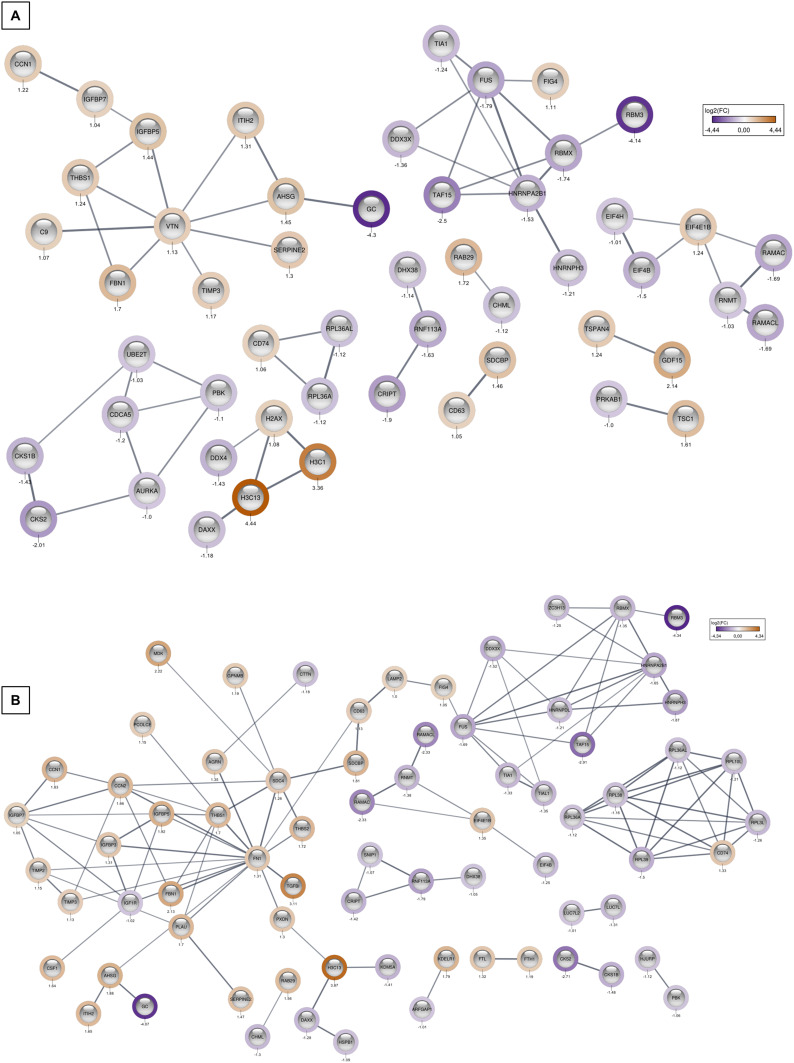
STRING/Cytoscape Network of the statistically significant changed proteins in T98G after treatment with ND (**A**) and NDM (**B**). Upregulated proteins are marked with an orange ring and downregulated proteins are marked with a purple ring; color intensity correlates with the fold change of the protein. Proteins are labelled with their gene name and log2FC value.

Upregulated proteins that were significantly enriched in both groups were involved in response to Ca^2+^ ionic imbalance and focal adhesion-related PI3K/Akt/mTOR pathway (Figure 9A and C). Detected pathways linked to downregulated proteins were associated with RNA processing, RNA splicing, translation initiation, and the regulation of these processes in both treatment variants (Figure 9B and D). Additionally, proteins linked to IGF transport and uptake, ECM interactions and post translational phosphorylation were augmented specifically in ND group, while ND elevated group of proteins involved in Senescence Associated Secretory Phenotype (SASP). A detailed presentation of proteins assigned to particular categories, as well as principal component analysis (PCA) of the proteomics results for T98G cells, is provided in the Supplementary Data (Figures S3, S4 and Table S2). Using STRING software, 10-protein interaction networks were created from a pre-selected list of proteins, ranging from 2 to 12 proteins that were directly or indirectly functionally connected (Figure 10A). A corresponding list of networks was created for the NDM-treated group, containing networks composed of 2 to 45 physically and functionally connected proteins (Figure 10B).

Among the networks with mostly downregulated proteins, one cluster involved RNA-binding proteins surrounding FUS (fused in sarcoma) protein. This group included cytotoxic granule-associated RNA-binding protein (TIA1), ATP-dependent RNA helicase (DDX3X), TATA-binding protein-associated factor 15 (TAF15), heterogeneous nuclear ribonucleoproteins A2/B1 (HNRNPA2B1), H3 (HNRNPH3), RNA-binding motif protein X chromosome-linked (RBMX, also known as HNRNPG), RNA-binding motif protein 3 (RBM3), and polyphosphoinositide phosphatase (FIG4), which was the only upregulated protein within this network. Following NDM treatment, two additional proteins in this cluster, TIA-like 1 (TIAL1) and zinc finger CCCH domain-containing protein 13 (ZC3H13), were downregulated. Another protein network with the highest abundance-linked histones H2AX (H2AX) and two histone H3 family members (H3.1 (*H3C1*) and H3.2 (*H3C13*) with death-associated protein 6 (DAXX) and DEAD-box helicase 4 (DDX4). In the NDM-treated group, only H3.2 overexpression was observed among histones. Another altered protein group in NDM-treated cells consisted of ribosomal proteins RPL3L, RPL10L, RPL36A, RPL36AL, RPL38, and RPL39, which were linked to the highly abundant cluster of differentiation 74 (CD74). In contrast, ND treatment only altered two of these ribosomal proteins: RPL36A and RPL36AL. Both types of diamond nanoparticles upregulated small GTPase RAB29 while simultaneously reducing CHM-like Rab Escort Protein (CHML). PDZ binding kinase (PBK) levels were also decreased following both treatments. However, NDM caused a reduction in only one Holliday junction recognition protein (HJURP) interacting with this kinase, whereas ND decreased five additional proteins (Aurora kinase A [AURKA], Cyclin-dependent kinase regulatory subunits [CKS1B, CKS2], Cell division cycle associated 5 [CDCA5], ubiquitin [Ub]-conjugating enzyme E2T [UBE2T]). Another network regulated by nanoparticle treatment involved RNA methyltransferase RNTM and its regulatory subunits RAMAC and RAMACL, as well as eukaryotic translation initiation factors EIF4B, EIF4H, and EIF4E1B. The EIF4E1B gene product was upregulated in both treatment variants. The abundance of EIF4H was downregulated in both groups but significantly altered only by ND.

On the other hand, NDM decreased RAMAC/RAMACL levels five-fold, whereas ND caused a three-fold reduction. Other interaction networks affected by NDMs included splicing factors LUC7 family members (LUC7L, LUC7L2), protein tyrosine C-terminal Src kinases (CSK2, CSK1B), Arf GTPase-activating proteins (ARFGAP1), and its receptor (KDELR1), as well as networks consisting of cysteine-rich PDZ-binding protein (CRIPT), Smad nuclear interacting protein 1 (SNIP1), E3 ubiquitin-protein ligase (RNF113A), and ATP-dependent RNA helicase (DHX8). The largest networks of mostly upregulated proteins in both cases were linked to extracellular matrix components, including vitronectin (VTN) in ND-treated cells and fibronectin 1 (FN1) in NDM-treated cells. ND-treated samples showed increased levels of tissue inhibitor of matrix metalloproteinases 3 (TIMP3), fibrillin 1 (FBN1), thrombospondin 1 (THBS1), complement component C9 (C9), insulin-like growth factor-binding proteins 5 and 7 (IGFBP5, IGFBP7), cellular communication network factor 1 (CNN1), glia-derived nexin (SERPINE2), and serum proteins such as α-2-HS-glycoprotein (AHSG) and inter-α-trypsin inhibitor protein (ITIH2). In the NDM group, the number of upregulated proteins was overall higher. Additional upregulated proteins included tissue inhibitor of matrix metalloproteinases 2 (TIMP2), insulin-like growth factor-binding protein 3 (IGFBP3) with a simultaneous decrease in IGF1R expression, colony-stimulating factor 1 (CSF1), cellular communication network factor 2 (CNN2), peroxidasin/vascular peroxidase-1 (PXDN), procollagen C-endopeptidase enhancer (PCOLCE), agrin (AGRN), syndecan 4 (SDC4), urokinase (PLAU), and lysosome-associated membrane protein 2 (LAMP2). Transforming growth factor-beta-induced protein ig-h3 (TGFB1), CD63 molecule (CD63), syndecan-binding protein/syntenin 1 (SDCBP), thrombospondin 2 (THBS2), transmembrane glycoprotein Nmb (GPNMB), and midkine (MDK) were also significantly increased in ND-treated cells but to a lesser extent. A striking finding was the over twenty-fold decrease in vitamin D-binding protein, encoded by the GC gene, after both treatments. The complete network structure is presented in Figure 9.

Statistically significant differences for NDM comparing to ND group were observed for 12 proteins that were upregulated: keratin 1 (KRT1), laminin subunit gamma-3 (LAMC3), SEC14 domain and spectrin repeat-containing protein 1/SOLO protein (SESTD1), DDB1 And CUL4 Associated Factor 11 (DCAF11), sororin (CDCA5), calcium-responsive transactivator-CREST protein (*SS18L1*), Adhesion molecule with Ig like domain 2 (AMIGO2), Actin-binding LIM protein 3 (ABLIM3), protein kinase CAMP-activated catalytic subunit gamma (PRKACG), trinucleotide repeat containing adaptor 6B (TNRC6B), and two cyclin-dependent kinase 2-associated proteins (CDK2AP1, CDK2AP2). NDM also caused prominent downregulation comparing to ND treatment of 10 proteins: transgelin-3 (TAGLN3), BMP-2-inducible protein kinase (BMP2K), tyrosine kinase receptor IGF1R (IGF1R), ankyrin-2 (ANK2), FKBP prolyl isomerase 1C (FKBP1C), apolipoprotein 3 (APOL3), RAB GTPase Activating Protein 1 Like (RABGAP1L), transporter protein solute carrier family 35 member B4 (SLC35B4), Ubiquitin Conjugating Enzyme E2 F (UBE2F), and Transcription Factor 7 Cofactor (MLLT11).

## Discussion

Physicochemical characterization of diamond nanoparticles revealed a typical tendency toward aggregation, which, to some extent, can be restrained by simple sonication.^41^ A strongly cationic Zeta potential (ZP) of around 30 mV and a PDI below 0.3, which is considered satisfactory for nanoparticles used as molecular carriers, was demonstrated only by colloids in water.^42^ The stability of these nanoparticles changed drastically when added to the cell culture medium due to a well-known protein corona formation process.^43^ In in vitro studies, nanoparticles are usually dispersed in standard culture medium supplemented with FBS or in serum-free medium.^44^ As previously demonstrated, exposure to nanoparticles in serum-free conditions can enhance nanoparticle adhesion to the cell membrane, intensify endocytosis, and, in some cases, lead to an increased cytotoxic effect.^45–47^ The ZP values of nanoparticles in FBS-free culture medium were just below −10 mV, indicating weak anionic properties, while the ZP values of NDs in FBS-supplemented medium remained closer to neutral.^41^ Using FBS during treatment with diamond nanoparticles can influence the cellular response due to the protein corona formation on the nanoparticle surface.^48^ This phenomenon is, however, considered beneficial for stability of dispersed nanoparticles in culture media because the outer protein layer restricts uneven aggregation in salts-rich environment.^49^ On the other hand, the cellular response may be influenced by the ability of cells to endocytose large complexes and variations in the specific components present in the medium.^50^ The impact on nanoparticle biocompatibility and subcellular target specificity may be determined not only by the presence of the protein corona but also by its size and composition.^51^ In many cases, the serum protein corona serves as a favorable component on the NDs surface, enhancing their biocompatibility.^44^,^52^ However, our previous research demonstrated concentration-dependent minor toxicity of diamond nanoparticles toward the T98G cell line in FBS-supplemented medium, as assessed by the LDH leakage assay.^53^ Conversely, in the present study, under completely serum-free conditions, NDs caused no membrane damage and did not impact the metabolic state of the cells. A notable limitation of this approach lies in the challenge of maintaining a stable dispersion of nanoparticles. Media components such as vitamins, carbohydrates, and ions promote aggregation and determine the charge of the outer layer.^54^

Nanoparticles with a lower sp² surface carbon content, which was demonstrated by the analysis of sp²-related G-bands in Raman spectral analysis in our previous work,^33^ exhibited prolonged hydrocolloidal stability. Only the NDM hydrocolloid maintained stable ZP values over time.^55^ This difference, however, was completely eliminated upon contact with the culture medium, suggesting that while the sp²/sp³ carbon ratio plays a critical role in the composition and stability of the protein corona, which is essential for biological applications, it is not the sole determinant of whether the corona forms.^48^ Despite the high rate of aggregation and changes in the ionic properties of both NDs in culture medium compared to water, SEM images revealed a high affinity of the aggregates for the cellular membrane, while optical microscopy observations and cytotoxicity assays confirmed their effective internalization within cells without compromising cell viability. Based on these findings, the next step was to investigate potential alterations in membrane-dependent processes, including early adhesion and motility.

The T98G glioblastoma cell line has been characterized as morphologically heterogeneous and resistant to standard temozolomide treatment. It exhibits high expression levels of O6-methylguanine methyltransferase (MGMT) and harbors mutations in tumor suppressors PTEN on chromosome 10 and TP53, as well as in the telomerase reverse transcriptase (TERT) promoter. According to the most recent WHO classification of central nervous system tumors, these characteristics, combined with an IDH wild-type status and pro-angiogenic potential, establish T98G as a reliable glioblastoma model, particularly for studying therapy resistance.^1^,^56–58^ Glioblastoma cells exhibit high motility, actively invading the surrounding environment, a process triggered by chemical signaling and mechanosensing.^59^ The primary mechanism that activates this migratory state is the epithelial–mesenchymal transition (EMT). However, in collectively migrating cells, the shift to a mesenchymal phenotype varies among individual cells within the cluster.^60^ Leader cells exhibit a characteristic mesenchymal phenotype, with pronounced polarity, enriched stress fibers, and overexpression of ECM-degrading proteins while following cells demonstrate so-called hybrid EMT state, maintaining some of the epithelial traits and stronger intracellular connections.^61^ This variability is particularly relevant for nanoparticle-based therapies as cellular signaling and responses to nanoparticles may differ depending on phenotypic differences and variations in nanoparticle exposure levels among migrating cells.^28^ To assess these differences, time-lapse imaging was performed on low-density cell cultures to examine the effects of NDs on individual migrating cells, while the exclusion zone migration assay was employed to evaluate collective migration patterns.^62^ The effects of diamond nanoparticles on cell motility appear to be dependent on both cell phenotype and nanoparticle properties. Previously, functionalized NDs have been shown to reverse the EMT in HeLa and breast cancer cells.^63^,^64^ The 70 nm fluorescent high-pressure high-temperature (HPHT) NDs used by Reyes-San-Martin et al did not affect HeLa cell motility at concentrations up to 100 µg/mL.^65^ In this study, NDs did not significantly alter the spontaneous migration of individual cells within the first 24 hours of exposure and promoted new tracks formation during the latter half of the incubation period. This stimulation may be connected with intense endocytosis of NDs stimulating the metabolic activity as a stress response. However, both NDs disrupted the migrating front and slowed the leader cells in migrating collectives during prolonged incubation. Interestingly, leader cells demonstrated a higher capacity for endocytosis as they appeared visibly more loaded with nanoparticles than the second-row cells. Moreover, the nanoparticle layer formed in the cell-free space between migrating cells could serve as an additional mechanical barrier further influencing migration dynamics. Although previous studies using different methodological approaches – where U87 and U118-MG cells were treated prior to insert removal – demonstrated a more pronounced inhibitory effect on migration, reducing cell-covered areas by at least two-fold after 48 hours of incubation.^27^

Due to the complex and multifaceted interactions of diamond nanoparticles (NDs) with cells – mediated by endocytosis, intracellular signaling regulation, interactions with membrane receptors, and mechanotransduction – the dynamics of early cell adhesion to different surfaces, including ECM components, was evaluated. Previous studies have demonstrated that ND-coated surfaces exhibit pro-adhesive properties when in contact with polystyrene and ECM components, including collagen type I, which was used as a representative ECM protein in this study.^53^,^66^,^67^ Exposure to the colloidal form of NDMs led to a significant reduction in cell adhesion, which was accompanied by the blockade of all studied integrins, while NDs exhibited higher binding affinity toward α subunits. This outcome explains the restricted adhesion observed, despite α1 integrin, a primary collagen-binding subunit, being the most highly expressed.^68^

Integrins, as transmembrane receptors, serve as a bridge between the intracellular environment and the ECM.^69^ Among the 18α and 8 β known integrin subunits, adhesion via 11 individual subunits and 3 heterodimers was analyzed in this work. Considering integrins that are highly expressed in gliomas and are critical for tumor survival, progression, and angiogenesis, NDM exhibited a stronger blocking effect than NDs, particularly against αVβ5, α5β1, and other αV, α5, β4, and β1 subunits.^68^,^70^ In glioma cell lines, blocking α3β1 integrin binding sites with antibodies significantly reduced migration and invasion in U251 glioma cells inoculated into nude mouse brain tissue.^71^ Moreover, α5, αV, and β1 integrins, which contribute to laminin and RGD motif receptors – including ligands such as vitronectin and fibronectin – are often highly abundant in metastatic, treatment-resistant cancer cells, and are considered potential therapeutic targets.^69^,^72^,^73^ Gao et al demonstrated that carboxylated diamond nanoparticles inhibit integrin β1 mRNA expression after 24 hours of exposure.^74^ Differences in nanoparticle effects on adhesion-related proteins may stem from their physicochemical properties and sp² carbon ratio as the graphitic layer could influence binding efficiency.^75^ Another key integrin affected by both types of diamond nanoparticles was αVβ3, which is widely studied as a molecular target for high-grade glioblastoma.^76^ In a protocol demonstrated by Neburkova et al, fluorescent nanodiamonds with a polymer shell were coated with RGD peptides and successfully used for U87 MG tracking via αVβ3.^77^ Here, pure diamond nanoparticles effectively bound several integrins with high affinity. Moreover, blocking αVβ3 and αVβ5 integrins has been consistently shown to be an effective strategy for enhancing the efficacy of therapeutic agents in glioma cells.^78^ In some cases, simple αV knockdown can activate alternative pro-survival pathways, such as EGFR-mediated Akt signaling, to overcome this blockade.^11^ Since NDs preferentially bound to the most abundant integrins, whereas NDMs exhibited affinity for a broader spectrum, including all tested β subunits, they may represent more versatile vectors for integrin-targeted therapeutic approaches.

Integrin α5β1 plays a key role in retraction fibers (RF) and migrasome formation, both involved in guiding cell migration. RFs are actin-based remnants at the cell’s trailing edge, whereas migrasomes are EV-like organelles that accumulate at RF intersections.^79^,^80^ The extensive coverage of RFs by NDs, clearly visible in SEM images, along with the high affinity of NDs for α5β1, may have contributed to slower migration in follower cells and enhanced local adhesion site strength.

Exploring the intracellular effects of integrin blockade, we conducted a comprehensive proteomic analysis to assess the impact on intracellular signaling and to identify potential integrin-independent regulatory pathways influenced by NDs. To ensure accurate gene ontology (GO) analysis, highly abundant serum proteins – albumin (ALB), hemoglobin subunits (HBA1, HBB, HBD), and α-2-HS-glycoprotein (AHSG) – were excluded to prevent masking effects on less abundant proteins. Their presence likely originated from residual FBS, as confirmed by Hemelaar et al, who identified these proteins as predominant components in the protein corona of FBS-incubated nanoparticles.^49^

One of the main networks altered by NDs centered around the DNA/RNA-binding protein FUS, a member of the FET (FUS/EWS/TAF15) family. FUS and TAF15 share functional similarities in RNA binding, transcription, and alternative splicing regulation. They are predominantly localized in the nucleus but can also be observed in stress granules following oxidative stress exposure.^81^ TIA1, a well-established stress granule marker, was also affected.^81^ Overexpression of FUS in high-grade gliomas plays a crucial role in tumor progression and malignancy by influencing multiple regulatory pathways.^82^,^83^ Destabilization of FUS-mediated signalization repeatedly led to inhibition of glioblastoma cells migration, invasiveness, drug resistance, and glioma angiogenesis.^84^,^85^

RNA-binding proteins (RBPs), due to their extensive involvement in post-transcriptional regulation, are multifunctional molecules that can act as either tumor suppressors or oncogenes in different cancers.^86^,^87^ Some RBPs are produced as a downstream response to cellular stress, which may contribute to pro-survival processes and drug resistance.^81^,^88^,^89^ Multiple RBPs with documented oncogenic activity specifically in glioblastoma were significantly downregulated following ND treatment, including HNRNPA2B1, both homologs of DDX3 helicase, RBM3, and RBM6.^87^,^90–93^ Downregulation of these proteins in other studies suppressed the EMT, proliferation, and survival of the cells by altering multiple signaling routes, like STAT3, PI3K/Akt pathways, influencing DNA breaks repairment and maintenance of genome stability.^91^,^94^,^95^ In complementary, other carbon nanomaterials were found to decrease the phosphorylated STAT3 levels, but only in highly invasive glioblastoma cell line.^96^ Carbon nanoparticles, including NDs, demonstrated also a reduction in U87 and U118 cells invasiveness, which was accompanied by downregulation of phosphorylated Akt and mTOR kinases.^27^ Our findings did not reveal significant alterations in these signaling kinases or transcription factors. However, the observed reduction in migration, adhesion, and the downregulation of regulatory RBPs, STAT3 effectors such as cortactin (*CTTN*, Table S1), an actin-binding protein involved in invadopodia formation, as well as αN-catenin (*CTNNA2*, Table S1) – the structural linker of cadherin and catenin complexes with cytoskeleton – aligns partially with previous findings on carbon allotrope nanoparticles.^97^,^98^ This suggests that NDs exert a secondary multi-targeting effect through the regulation of multiple RBPs.

The methyltransferase RNMT/RAMAC complex is required for efficient RNA capping, catalyzing the methylation of the guanosine cap at the N7 position, converting it into 7-methylguanosine (m7G) – a crucial modification in RNA polymerase II-transcribed RNA.^99^ Another m7G modification regulator, MTTL1, has been associated with drug resistance in various cancers and is found to be upregulated in gliomas.^100^ Although the precise role of the RNMT/RAMAC complex remains unclear, based on previous reports, its downregulation may be a beneficial outcome in the context of glioma treatment response. Conversely elevated EIF4E1B after NDs treatment was found to have a negative correlation with other m7G methylation regulators and was designated as a favorable prognostic factor for glioblastoma patients.^101^ Proteins involved in RNA transport and stability, translation, splicing regulation, and miRNA sorting into extracellular vesicles are considered novel but attractive molecular markers or potential therapeutic targets.^87^,^102^ Nevertheless, their role in tumors can drastically vary and needs further investigation, especially in highly heterogenous tumors, like glioblastoma.

Moreover, among proteins altered after NDs treatment were ribosomal proteins (RPL). Essential for assembling the large (60S) ribosomal subunit, RPLs play a complex role in ribosome biogenesis, translation, and beyond. Their deregulation can induce secondary transcriptomic changes, impacting cell growth, responsiveness to external stimuli, and positioning specific RPLs as either tumor suppressors or oncogenes.^103^ One of the key correlations between glioblastoma prognosis and RPL involved RPL39, which has been identified as a prognostic factor due to its overexpression in glioma patients and cell lines. Moreover, in vitro studies demonstrated that RPL39 knockdown reduced glioblastoma cell proliferation, migration, and colony formation while inhibiting M2 macrophage infiltration.^104^ Similarly, knockdown of RPL36A in oral cavity squamous cell carcinoma enhanced cell sensitivity to DNA damage and radiation-induced apoptosis.^105^ Despite these findings, the ribosomal machinery exhibits extreme heterogeneity across tissues, making the precise role of RPL proteins in glioblastoma signaling pathways yet to be fully resolved.

Additional network strongly affected by ND consisted of AURKA, CKS1B, CKS2, CDCA5, UBE2T, and PBK, which was the only one downregulated by both NDs. Above proteins are upregulated in glioblastoma, involved in cyclin-dependent kinase regulatory activity, prooncogenic pathways (PI3K/Akt, WNT/β-catenin, TGFβ), migration, invasion, cell cycle progression, and therapy resistance; thus, they are widely explored therapeutic targets.^106–110^

Among the upregulated proteins, several exhibit tumor-suppressive potential. NDs upregulated hamartin (*TSC1*), which has documented neuroprotective and tumor suppressing properties and negatively modulates pro-survival mTOR pathway.^111^ Additionally, NDM significantly elevated WNT/β-catenin downregulator – Dickkopf-related protein 3 (*DKK3*).^112^ NDs modulated the expression of proteins directly and indirectly involved in the Hippo/YAP signaling pathway. Specifically, ND treatment increased levels of cysteine-rich 61 (CYR61, *CCN1*), NDM upregulated connective tissue growth factor (CTGF, *CCN2*) whereas both NDs elevated coiled-coil domain-containing protein 80 (CCDC80) levels. Although their role in glioblastoma remains unresolved, research on colorectal and thyroid cancer cells suggests that CCDC80 acts as a tumor suppressor by inhibiting anchorage-independent growth and malignant characteristics.^113^,^114^ It was also connected with WNT/β-catenin pathway.^115^ CCN family members appear to have more diverged roles. CCN1 is linked with more invasive phenotype in many tumors, including glioblastoma, while reports involving CNN2 indicate that CNN2 function may be cell-type specific.^116^ A noteworthy study conducted by Lee et al on T98G glioblastoma cells demonstrated that knockdown of the tumor suppressor neurofibromatosis 2 (NF2) led to an upregulation of both CNN proteins. Further silencing of CYR61/CCN1 reduced glioblastoma invasion, whereas knockdown of CTGF/CCN2 enhanced the invasive potential of T98G cells.^117^ Both NDs caused a significant elevation of protease nexin 1 (PN1, *SERPINE2*), also known as glia-derived nexin, which has been reported to have an inhibitory effect on tumor migration in rat C6 glioma cells.^118^ Another key finding was the nearly four-fold increase in protein kinase Cγ (PRKCG), which is regulated by intracellular Ca²⁺ levels and is often downregulated or hypermethylated in high-grade gliomas – an alteration strongly correlated with poor survival.^119^ Previous studies by Kumari et al demonstrated that carboxylated NDs induce increased intracellular Ca²⁺ and ROS levels in platelets, leading to their activation.^120^ Similarly, Setyawati et al suggested that increased Ca²⁺ and ROS contribute to the enhanced permeability of the endothelial barrier in ND-treated cells.^120^

According to DAVID classification, numerous of the upregulated proteins after NDs treatment are associated with poor glioblastoma prognosis, ECM remodeling, cell motility regulation, PI3K-Akt pathway activation, and the senescence-associated secretory phenotype (SASP). Among them, cyclin-dependent kinase 4 inhibitor B (CDKN2B) is correlated with SASP cytokine secretion in colorectal and liver cancers.^121–123^ Nevertheless, in glioblastoma patients, deletion of CDKN2A/B directly corresponds with worse overall survival rates. Additionally, several upregulated proteins, including CD74, GDF15, IGFBP3, IGFBP5, and IGFBP7, are associated with higher glioblastoma grade and immune infiltration.^124–129^ Tumor microenvironment proteins such as thrombospondins (THSPs), vitronectin (VTN), fibronectin (FN1), and midkine (MDK), which promote the PI3K/Akt pathway, were also significantly upregulated.^130^ A similar phenomenon of increased ECM protein production, including fibronectin, collagen, vitronectin, and vimentin, has been reported for other carbon-based nanomaterials, such as single-walled carbon nanotubes or graphene.^131^,^132^ The upregulation of these proteins, typically accompanied by increased expression of matrix metalloproteinases facilitating ECM remodeling, has been associated with the induction of epithelial-to-mesenchymal transition (EMT).^131^ The elevated abundance of canonical ECM proteins, such as fibronectin, may also represent an early phase of the foreign body response (FBR), a well-documented process in the context of biomaterials, whereby protein adsorption stimulates ECM production and subsequently triggers inflammatory cascades leading to fibrosis.^133^ However, the absence of concomitant upregulation of MMPs, collagens, or vimentin and others EMT markers in our study does not support the promotion of a pro-inflammatory pro-migratory phenotype.^131^ The intriguing difference between treatment variants was significantly higher abundance of vitronectin in NDs treated group and fibronectin in NDM group. According to Johnson et al, remodeling the microenvironment composition can significantly influence the astrocytes response to stimuli. The team demonstrated that the migration of primary astrocytes after performing the scratch assay would be restricted on fibronectin surface and promoted on surface covered with vitronectin or tenascin. On the other hand, cell cultured on fibronectin would be more responsive to inflammatory stimulation. The cellular response would also be mediated by integrin β1.^134^ Interestingly, while several upstream components of the PI3K/Akt/mTOR pathway were elevated; downstream effectors such as translation initiators EIF4B,^135^ EIF4H,^136^ and IGF1R^137^ were downregulated. Notably, IGF1R levels differed drastically between treatment variants, with downregulation observed only in NDM group, highlighting potential differences in molecular responses to ND and NDM treatments.

Proteome analysis also identified several proteins involved in extracellular vesicle (EV) formation. Upregulated EV markers included syndecan (which co-expresses with syntenin for exosome biogenesis),^138^ tetraspanin-4 (a migrasome-specific marker), CD63 (an exosome marker), and lysosomal LAMP2, which modulates exosomal cargo.^139^,^140^ Studies by Hallal et al revealed that EVs released by glioblastoma cells promote SASP activation in normal astrocytes, suggesting that stress-induced exosomal cargo may contribute to treatment resistance.^141^ EVs produced by glioblastoma under stress conditions induced by treatment contain numerous proteins involved in splicing machinery, such as HNRNPs. As a consequence, EV-recipient cells undergo several splicing alterations and acquire a malignant, resistant phenotype.^142^ Other significantly augmented proteins were canonical histones H3, which were previously found elevated in high-grade glioblastomas and stress-induced EVs.^143^ Interestingly, NDM downregulated histone demethylase KDMA5, which is considered treatment resistance inducer.^144^ This trend was followed by NDs but did not reach the threshold cutoff. Other studies’ results revealed that targeting KDM5A and other histone demethylases results in higher efficiency of cytostatics.^145^ Conversely, the upregulation of H2AX observed only in the ND group may be associated with stress-mediated DNA double-strand breaks (DSBs). Previous studies have demonstrated higher ROS levels in T98G cells treated with ND compared to surface-purified ND-COOH (NDM), which are recognized inducers of cellular stress.^33^ In the study by Liu et al, a positive correlation was reported between H2AX protein-level abundance and sensitivity to chemotherapeutic agents, with the levels of total and phosphorylated protein were increasing proportionally.^146^ Nonetheless, phosphorylated H2AX is a well-established DSB marker that coordinates the recruitment of repair machinery, whereas an increase in total H2AX levels does not have such a clear prognostic value.^147^ Proteome analysis following NDs treatment revealed an enriched presence of exosomal markers, although the presence of EV and their cargo need further investigation. However, the majority of splicing-related proteins, often released as a stress response, were strongly downregulated.^142^

The role of TIMPs and their MMP ligands in glioblastomas remains unclear as reported results are inconsistent. In general, higher expression of TIMP3 is associated with a more favorable prognosis in glioblastoma patients.^148^ TIMP2 can be upregulated under stress conditions. It has various functions and, apart from MMP2, interacts with numerous proteins to regulate ECM remodeling and cellular signaling. Moreover, it demonstrated antiangiogenic and antimigrative activity as an antagonist of IGF-1R and through α3β1 signaling.^149^,^150^ Notably, TIMP3 expression was upregulated following treatment with both NDs. While TIMP2 levels reached the threshold cutoff only in the NDM group, a similar trend was observed in the ND group. Taheri et al revealed that exogenous TIMP3 had no impact on the proliferation of T98G cells but significantly reduced their migration rate.^151^ Wen et al achieved successful MMP2 depletion, decreased tumor volume, and prolonged survival in mice with U87MG-induced glioblastoma using a bacterial vector for controlled TIMP2 expression.^152^ In summary, the upregulation of TIMP2 and TIMP3 could serve as an additional anti-migratory factor.

## Conclusion

This study contributes to understanding how the physicochemical surface properties of nanodiamonds—such as the presence of an sp² carbon layer—may influence their interactions with the glioma microenvironment by affecting protein binding, including key integrins at the cell surface, and potentially altering the cellular proteome. NDs reduce T98G glioblastoma cell adhesion and collective migration. Lower surface graphitic content enhances affinity to cellular adhesive proteins, particularly integrins that are considered crucial for tumor progression (αv, β1, β4, αvβ5, α5β1). Proteome analysis revealed that NDs treatment upregulated several proteins related to tumor ECM, such as FN1 and THSPs in the ND-treated group and VTN after NDM treatment, while key ECM components and stemness-promoting factors, including hyaluronan, collagens, and CD44 were not detected. By shaping the predominance of specific ECM proteins within the microenvironment, ND may influence ECM remodeling. Since nanoparticles with different surface properties can shape the ECM composition of the tumor microenvironment, by preferentially promoting the production of, and exhibiting higher affinity for, proteins like vitronectin or fibronectin, they may serve not only as carriers of active biomolecules but also as agents actively remodeling the tumor microenvironment. Advancing our knowledge of ECM dynamics under physiological and pathological conditions, supported by nanoparticles with known surface chemistry may facilitate the rational design of more effective therapeutic solutions.

In contrast, intracellular splicing regulators and RNA processing factors were significantly downregulated by both types of NDs. Proteome alterations suggest that, due to disruption of RNA processing balance and other factors (like high abundance of DSB marker after ND treatment) glioblastoma cells can become more responsive to treatment following NDs pretreatment. This study provides novel insights into the interactions of NDs with the glioblastoma environment and identifies promising molecular targets for future drug-delivery strategies, tumor monitoring, and therapeutic approach design. Nevertheless, protein targets detected and described herein require further investigation, as some have roles in glioma progression that are not yet fully understood or clearly defined. Future studies could examine protein corona composition and interactions in more complex microenvironments using highly purified NDs and diverse cellular models.

## Data Availability

The data are available from the corresponding author on reasonable request.
